# Media Reporting on Air Pollution: Health Risk and Precautionary Measures in National and Regional Newspapers

**DOI:** 10.3390/ijerph17186516

**Published:** 2020-09-07

**Authors:** Steven Ramondt, A. Susana Ramírez

**Affiliations:** 1 Department of Communication Science, Vrije Universiteit Amsterdam, 1081 HV Amsterdam, The Netherlands; 2 Psychological Sciences, University of California, Merced, CA 95344, USA; 3 Public Health, University of California, Merced, CA 95344, USA; sramirez37@ucmerced.edu

**Keywords:** air pollution, environment health, public health, newspapers, environmental health literacy, health promotion, health communication, efficacy, risk communication, advocacy

## Abstract

Exposure to air pollution is one of the primary global health risk factors, yet individuals lack the knowledge to engage in individual risk mitigation and the skills to mobilize for the change necessary to reduce such risks. News media is an important tool for influencing individual actions and support for public policies to reduce environmental threats; thus, a lack of news coverage of such issues may exacerbate knowledge deficits. This study examines the reporting of health risks and precautionary measures regarding air pollution in national and regional print news. We conducted a content analysis of two national and two local newspapers covering the USA’s most polluted region during a 5-year period. Coders identified information on threat, self-efficacy, protective measures and information sources. Nearly 40% of air pollution news articles mentioned human health risks. Fewer than 10% of news stories about air pollution provided information on the precautionary measures necessary for individuals to take action to mitigate their risk. Local newspapers did not report more threat (Χ^2^ = 1.931, *p* = 0.165) and efficacy (Χ^2^ = 1.118, *p* = 0.209) information. Although air pollution levels are high and continue to rise at alarming rates, our findings suggest that news media reporting is not conducive to raising environmental health literacy.

## 1. Introduction

Air pollution is the single largest environmental health risk and one of the largest global risk factors [1,2], with outdoor air pollution estimated to be responsible for almost 8% of total global deaths [3]. To reduce individual risk associated with air pollution, individuals need to be aware when air quality is poor [4,5,6]. The primary official forms of communication about air pollution to achieve this goal are air quality advisories; however, the information environment is much broader than targeted campaigns [7]. The broad public information environment is an important determinant of knowledge, attitudes, and other cognitive and emotional determinants of behavior [8,9,10], and should be investigated beyond air quality advisories, especially since awareness of air quality advisories often does not lead to behavior change, and air quality advisories are among the least reported sources of information on air pollution [4,11]. Research has found that media, together with sensory and health cures, are the primary sources of information in air polluted regions [11,12]. Information found in the media can increase awareness and change perceptions of environmental risks, such as air pollution, and help individuals with processes that lead to risk-reducing behavior [13,14]. Moreover, consistent with an ecological approach to health [2], recent research in environmental health literacy argues that messaging must move beyond exclusively focusing on individual behavior change to include strategies that empower individuals to mobilize for the control of environmental exposures [15,16,17]. Media influences which issues the public are exposed to and thereby sets the public agenda [18]. Agenda-setting research has furthermore shown that news coverage plays a role in shaping public opinion and the local policy agenda, and that this role is more prominent for local-level news [19].

The current research explores how air pollution is covered in news media in accordance with Wardman’s [20] instrumental imperative. We examine risk communication as a resource to change behavior in accordance with recommendations from health officials during episodes of poor air quality [21]. To investigate how messages can change individual behavior, we utilized the extended parallel process model (EPPM) [22]. The EPPM is commonly used to explain how individuals process health messages, and proposes that for an individual to accept a message and change their behavior, two appraisal steps are necessary. First, an individual needs to perceive a threat to themselves that warrants action, and second an individual needs to perceive themselves as able to avert the threat [22]. For an individual to rake risk-reducing action, they need to know about both the risk and effective actions they can take to reduce risk. We therefore conducted a content analysis of newspapers, and examined how individuals might process media messages by analyzing how much health risk (threat) and precautionary measures (efficacy information) information air pollution coverage contains. The effective precautionary measures individuals can take to reduce risk from outdoor air pollution include the following: staying indoors, limiting physical activity, or using air filters to clean indoor air during severe air pollution days [6]. However, it is important to note that while risk reduction is desirable, complete reduction of risk is implausible. In addition, precautionary measures can have downsides, including increasing air pollution risk. For example, some air filter cleaners produce ozone, acerbating air pollution risk [23]. Moreover, indoor air pollution can also be a major risk to public health, especially in developing countries [24]. In addition to examining health risks and information about precautionary measures, we examined the potential influence of journalists’ information sources on the framing of air pollution. The manner in which issues are presented or framed in the media affects the perceptions of the public [25]. Media coverage of environmental issues has been critiqued for lacking substance, adequate coverage and potential solutions [26,27]. The choice of source for a story influences how a story is framed, the substance that is included, and which solutions are provided [28].

National news was compared with local news from California’s San Joaquin Valley (SJV). The SJV is a rural and economically disadvantaged region that lacks resources and access to address environmental and public health threats [29,30]. Moreover, this region is one of the worst air polluted areas in the US [31]. Latino, low-income and less-educated populations—which are overrepresented in the SJV—have less access to health information [32,33]. For minorities that suffer from this lack of access, news media is the primary and most trusted source of health information [34]. News media, especially local news media, may be a particularly important source of information for residents of the SJV, since the lack of resources and the geographically-dispersed nature of rural areas such as the SJV make it hard to reach the population through other channels.

According to Ropeik and Slovic [35], effective risk communication requires more than merely the sending of the message; factors that shape individuals’ risk perceptions should also be taken into account. By assessing which essential risk-reducing information is missing in newspaper coverage, and which messages individuals are exposed to, health promotion efforts can be tailored, creating more effective campaigns. Consistent with prior content analyses of environmental risks [36,37], we hypothesized that (1) newspapers are more likely to report information about threats to health compared to efficacy information to reduce individual health risk. Due to the impact air pollution has on the SJV and the concerns it raises with its residents [38,39], we expected local newspapers to focus more on reporting about air pollution and reducing the adverse effects of air pollution. Therefore, we hypothesize that (2) local newspapers are more likely, compared to national newspapers, to report on the threat of air pollution to health, (3) local newspapers are more likely, compared to national newspapers, to provide efficacy information about precautionary measures individuals can take to reduce the risks of air pollution, and (4) local newspapers are more likely, compared to national newspapers, to report on three precautionary outdoor air pollution measures: staying indoors, limiting physical activity, and using air filters [6].

## 2. Materials and Methods

### 2.1. Study Sample

Two national newspapers, the New York Times and the Washington Post, were selected to represent the national-level discourse on air pollution in the media. Two newspapers from the SJV, the Fresno Bee and the Bakersfield Californian, represented local news about air pollution. The New York Times and the Washington Post have high circulation and influential status and are considered to be agenda setters for other media outlets in the US [18]. Both the Fresno Bee and the Bakersfield Californian are among the highest circulating papers in California’s air polluted San Joaquin Valley, and are the hometown papers of the two most polluted cities in the US [31].

### 2.2. News Coverage Selection

The data for this study were news stories about air pollution published in the four newspapers during the five-year period 2011–2015. News stories were obtained from the Lexis-Nexis database for the two national newspapers and the Newsbank World News database for the two local newspapers. Following procedures described by Stryker and colleagues [40], a search term was constructed. News stories about air pollution were operationalized as needing to include air pollution content in the title and/or first three paragraphs. The following search term was used to collect the sample: ATLEAST1 (air quality or air pollution) AND (air pollution or air quality or clean air or dirty air or polluted air or smok! or fume! or cloud or gas! or exhaust! or vapor or inhale! or breathe! or respir! or emission! or smog or ozone) in any of the first three paragraphs (HLEAD was used in Lexis-Nexis to automate this process). To keep our sample size manageable while obtaining an accurate estimate of the population, a constructed week sampling approach was used. Constructed week sampling is a stratified random sampling technique that is preferred to simple random sampling as it accounts for variation of news content over a seven-day news week [41]. The current study sampled 6 constructed weeks for each of the five years in which news stories (both national as well as local) were collected, for a total of 30 constructed weeks, yielding a total of 276 articles.

### 2.3. Measures

We measured threat, efficacy information and information sources. All measures were dichotomous items. Stories were coded as a threat if an article included any information about air pollution being adverse to health. Efficacy was coded if the article included *any* information about precautionary measures an individual can take to the reduce risks of air pollution. The coding of efficacy information included an additional stage. If an article included efficacy information, the nature of the efficacy information was investigated to see if the efficacy information included any of the effective precautionary measures individuals can take—staying indoors, limiting physical activity, or using air filters to clean indoor air during severe air pollution days [6]. To examine which sources were utilized in the articles about air pollution, 5 types of sources were coded. The source typology was based on work by Brossard and colleagues [42] and included academics and scientists, non-expert/citizen, business/industry groups, governmental sources and health, and environmental advocacy groups. All articles were analyzed to see if any of these sources were utilized. It was possible to code for multiple sources per article.

Once the coding instrument was developed, two coders were randomly assigned three sections (*n* = 109, 39.5% of total sample) to code. Cohen’s kappa showed substantial agreement (mean k = 0.68) [43]. The initial interrater reliability was below the threshold set a priori (k < 0.7) for three codes classifying cited sources: “non-expert/citizen sources”, “business/industry groups” and “health and advocacy groups”. To achieve a higher level of reliability, the two coders double coded all articles for these codes and conducted consensus meetings afterward. As a result, the final average Cohen’s kappa increased to a high agreement (mean k = 0.85) [43]. The remaining years of air pollution news articles were randomly distributed and coded independently by the two coders.

### 2.4. Data Analysis

To compare differences between local and national newspapers, chi-square independence tests were conducted. A Fisher’s exact test was used in case the expected cell count was less than 5. All descriptive statistics, reliability and chi-square tests were performed with IBM SPSS Statistics 24.0.

## 3. Results

A total of 276 articles met our selection criteria and were read and analyzed; this included 162 national newspaper articles and 114 local newspaper articles. The New York Times (*n* = 98) accounted for the majority of the coverage, followed by the Washington Post (*n* = 64), the Bakersfield Californian (*n* = 61) and the Fresno Bee (*n* = 53). There was no significant difference in the number of articles reporting on air pollution between local and national newspapers (Χ^2^ = 3.732, *p* = 0.053).

### 3.1. Threat and Efficacy

**Figure 1:**
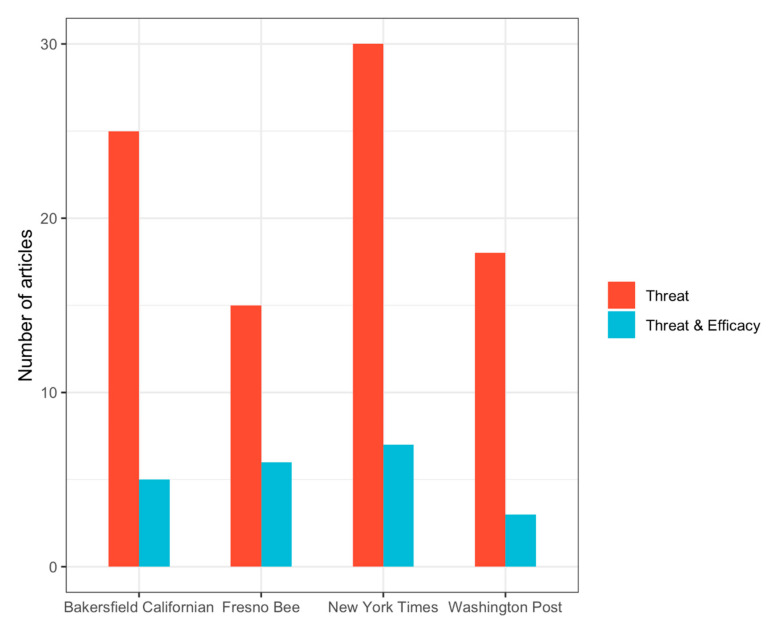
Threat and efficacy information per newspaper.

Threat information (39.9%) was reported more frequently than efficacy information (7.6%) in the combined sample (Χ^2^ = 34.626, *p* = 0.001). Threat was reported more frequently compared to efficacy in both local newspapers (Χ^2^ = 15.039, *p* < 0.001) and national newspapers (Χ^2^ = 18.935, *p* < 0.001). No newspaper reported efficacy information without reporting threat information (Figure 1).

Table 1 compares threat and efficacy information for local and national newspapers. When comparing local newspapers with national newspapers, local newspapers reported more threat information (44.7%) compared to national newspapers (36.4%). However, this difference was not statistically significant (Χ^2^ = 1.931, *p* = 0.165). Similarly, no significant difference (Χ^2^ = 1.118, *p* = 0.209) was found for the reporting of efficacy information in local newspapers (13.0%) compared to national newspapers (9.6%). When reporting recommended efficacy information, no significant differences were found for the individual risk-reducing behaviors “stay indoors” (Χ^2^ = 0.885, *p* = 0.347) or “use of air filters” (Χ^2^ = 0.953, *p* = 0.652). However, local newspapers did report more on “limiting physical activity” compared to national newspapers (Χ^2^ = 5.105, *p* = 0.036).

### 3.2. Information Sources

**Figure 2:**
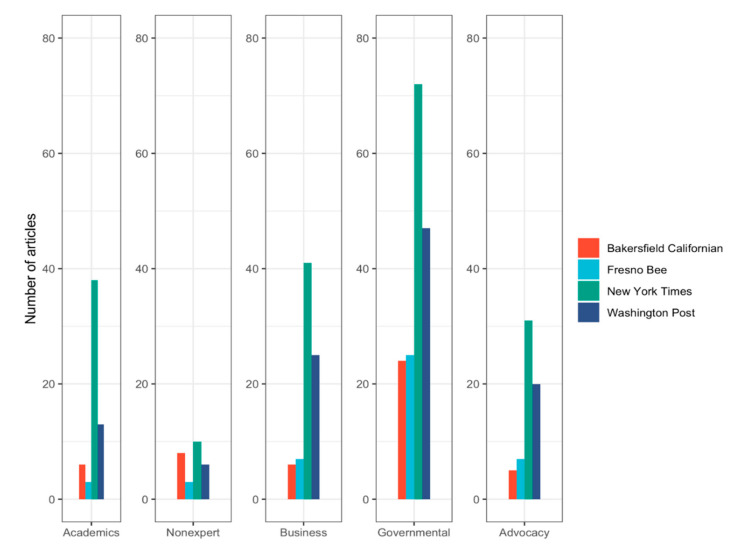
Sources utilized in local and national newspaper articles about air pollution.

Reporters primarily used governmental sources, followed by business/industry groups, health and environmental advocacy groups, academics and scientist, and non-expert/citizen sources (Figure 2).

A similar order was found for national newspapers. Local newspapers also used governmental sources primarily, followed by business/industry sources and health and environmental advocacy groups. However, they used more non-expert citizen sources compared to academic sources. National newspapers used disproportionally more information sources in their articles compared to local newspapers. As can be seen in Table 2, national newspapers utilized significantly more academic and scientific sources (Χ^2^ = 21.881, *p* < 0.001), business/industry groups (Χ^2^ = 28.189, *p* < 0.001), governmental sources (Χ^2^ = 26.089, *p* < 0.001) and health and environmental advocacy groups (Χ^2^ = 16.680, *p* < 0.001). No significant differences were found in the uses of non-experts/citizens as information sources by reporters (Χ^2^ = 0.004, *p* = 0.950).

## 4. Discussion

The present study looked at the nature of air pollution reporting in the media, exploring factors in news reporting on air pollution that might affect individual risk-reducing behavior. Similarly to other content analyses analyzing newspaper reporting of other health issues [37,44,45,46], we found that air pollution stories contained more threat information than efficacy information. It is important that newspapers report about the threat of air pollution to health, as this informs the public on the need for action. However, by not providing any information on what to do to reduce the introduced threat, undesirable side effects can arise. The EPPM [22] posits that when threat information is high and efficacy information is low, individuals will manifest a maladaptive coping response such as denial and avoidance of information [47]. While results can differ for individuals, as individual differences, including prior experiences, culture and personality, influence the appraisal of threat and efficacy [22], our results suggest that current reporting about air pollution in newspapers is not conducive to the promotion of risk-reducing behavior.

This study found that news reporting about air pollution lacked information about effective precautionary measures that individuals can take. Local newspapers in the SJV did not report significantly more about air pollution, threat and efficacy compared to national newspapers, despite being located in one of the worst air polluted areas in the USA, and even though air pollution is a major concern for residents of the valley [38,48]. The results are not entirely surprising, as the relative absence of news stories about air pollution is in line with a recent study analyzing local news reporting about health in the SJV, which also found limited coverage of air pollution [32]. However, the lack of efficacy in local publications is alarming as public information sources in the region have a similar deficiency [17], making it plausible that residents in the SJV have insufficient information available necessary to protect themselves from the adverse effects of air pollution. Public health advocates and health promotion experts must recognize the need to balance the structural causes of poor air quality and the actions individuals and communities can take to reduce air pollution-related morbidity and mortality. It is necessary to develop more effective strategies for disseminating information about the health risks of air pollution. National and local news media outlets may be useful partners for such dissemination, as media plays a vital role in the public understanding of environmental risks [49].

Similar to the content analyses of other environmental issues [27,42], both local and national newspapers over-relied on governmental sources. The high reliance on governmental sources is concerning, as they are likely to present established views [27]. Moreover, the high reliance on governmental sources is particularly concerning in the current political climate, as governmental agencies are acting in conflict with their goals. For example, the agency in charge of mitigating air pollution is advocating for relaxation of the Clean Air Act legislation [50]. The relative lack of sources that might present unconventional views limits the range of concerns and solutions presented in the news. This has implications for the policy changes necessary to reduce air pollution (health risk). For instance, changes in tobacco policy benefited from the voices of diverse groups and organizations in establishing public perceptions necessary to mobilize change. Collaboration in news media campaigns to increase the attention the media pays to diverse voices is therefore recommended [51]. Non-governmental groups, such as health and environmental groups and academics and scientists, should consider similar tactics to voice their concerns about air pollution. Academic and scientific sources were present in less than a quarter of the articles. The primary reliance of air pollution reporting on sources that might not be impartial and lack expertise might not be conductive to the understanding of air pollution by a general audience. Future studies should investigate if these sources cause environmental health literacy misinformation and misconceptions.

Despite its strengths, this study suffers from some limitations. To begin, only a select number of newspapers were included in the current study. It is possible that a selection of different newspapers would reveal different patterns. Similarly, a selection of different news sources (e.g., online news or broadcast) could show different results. However, we are reasonably confident that this is unlikely, because newspapers—and in particular widespread national newspapers, such as the national newspapers (i.e., New York Times and Washington Post) utilized in this content analysis—are agenda-setters for other media sources [9]. Our coding of threat and efficacy information used simple binary codes. The coding therefore ignores any nuanced tones and implications that potentially exist in the news story. Additionally, coverage patterns could have changed since the time of the study (2011–2015), as developments, such as the WHO campaign [52] to mobilize people to bring air pollution to safe levels, or the 2016 presidential election and resulting changes at the EPA, could have influenced the coverage. For example, the salient new efforts made by the WHO to convince the public and policymakers of the disastrous effects of air pollution by branding it “the silent killer” might have increased the amount of threat reporting in newspapers. We also did not investigate weather forecasts. Currently, weather reports can communicate threats from air pollution, but do not include behavioral recommendations. Hence, it is possible that the negative coping effects as postulated by the EPPM are more likely to happen. Lastly, our work analyzed air pollution communication using a top-down approach. This risk message model perspective [20], in which the audience passively receives information, is a simplified view of the communication process. While the message components we examined are essential requirements for individual behavior change, future research should take into consideration additional communication factors, including participatory communication components, that are salient for change [21].

## 5. Conclusions

The findings of this study suggest that reporting about air pollution in newspapers is not conducive to risk-reducing behavior. Newspapers mostly fail to report on the health impacts air pollution can have. Moreover, there needs to be a better balance between threat and efficacy information—especially effective precautionary measures that individuals can take—in the reporting about air pollution. Given the large impact air pollution has on the SJV, and the impact of local news on public opinion and the local policy agenda, more health-promoting news stories about air pollution would be beneficial. Health promotion efforts should consider the information in the media environment, and develop strategies to enhance the air pollution information environment. Health promotion efforts, such as the breath air campaign by the WHO, might mobilize people into action by increasing the amount of threat information available. However, to neutralize potential undesirable effects, campaigns would do well to provide efficacy information on how to reduce individual risk associated with air pollution. The current reliance on conventional sources of information by journalists might forestall the understanding of complex issues, such as air pollution. Air pollution reporting would benefit from more diverse, expert and impartial sources. News coverage of air pollution consistently misses opportunities to raise environmental health literacy. Health promotion efforts should consider using news media strategically to increase environmental health literacy.

## Figures and Tables

**Table 1:** Threat and efficacy information in news coverage of air pollution.

	Total (*n* = 276)	Local (*n* = 114)	National (*n* = 162)
Threat	110 (39.9%)	51 (44.7%)	59 (36.4%)
Efficacy	21 (7.6%)	11 (9.6%)	10 (6.2%)
Stay indoors	13 (4.7%)	7 (6.1%)	6 (3.7%)
Limit physical activity *	9 (3.3%)	7 (6.1%)	2 (1.2%)
Use air filter	5 (1.8%)	1 (0.9%)	4 (2.5%)

* Significant difference in amount of efficacy information about limiting physical activity between local and national newspapers, *p* < 0.05.

**Table 2:** Information sources cited in news coverage of air pollution.

Sources	Total (*n* = 276)	Local (*n* = 114)	National (*n* = 162)
Academics and scientist sources *	60	9	51
Non-expert/citizen sources	27	11	16
Business/industry groups *	79	13	66
Governmental sources *	168	49	119
Health and environmental advocacy groups *	63	12	51

Note: Each article can have multiple sources. * Significant difference in sources utilized between local and national newspapers, *p* < 0.05.
